# Disturbance of Shallow Marine Soft-Bottom Environments and Megabenthos Assemblages by a Huge Tsunami Induced by the 2011 M9.0 Tohoku-Oki Earthquake

**DOI:** 10.1371/journal.pone.0065417

**Published:** 2013-06-07

**Authors:** Koji Seike, Kotaro Shirai, Yukihisa Kogure

**Affiliations:** Atmosphere and Ocean Research Institute, University of Tokyo, 5-1-5 Kashiwanoha, Kashiwa, Chiba, Japan; Plymouth University, United Kingdom

## Abstract

Huge tsunami waves associated with megathrust earthquakes have a severe impact on shallow marine ecosystems. We investigated the impact of a tsunami generated by the 2011 M9.0 Tohoku-Oki earthquake on the seafloor and large benthic animals in muddy and sandy ria coasts (Otsuchi and Funakoshi bays) in northeastern Japan. We conducted underwater field surveys using scuba equipment in water depths of <20 m before the tsunami (September 2010) and after the tsunami (September 2011 and September 2012). During the study period, episodic changes in topography and grain-size composition occurred on the seafloor of the study area. Megabenthos sampling revealed a distinct pattern of distribution succession for each benthic species. For example, the protobranch bivalve *Yoldia notabilis* (Bivalvia: Nuculanidae) and the heterodont bivalve *Felaniella usta* (Bivalvia: Ungulinidae) disappeared after the tsunami event, whereas the distribution of the venus clam *Gomphina melanaegis* (Bivalvia: Veneridae) remained unchanged. In addition, the patterns of succession for a single species, such as the giant button top shell *Umbonium costatum* (Gastropoda: Trochidae) and the heart urchin *Echinocardium cordatum* (Echinoidea: Loveniidae), varied between the two bays studied. Our data also show that reestablishment of some benthic animal populations began within 18 months of the tsunami disturbance.

## Introduction

Huge tsunami waves associated with megathrust earthquakes have devastating effects on shallow marine ecosystems. For example, the 2004 Indian Ocean tsunami disturbed the benthic ecosystems of mangrove forests [1] and seagrass beds [2], [3] in the intertidal environments of the Indian Ocean and Andaman Sea. Tsunami generated by the 2010 M8.8 earthquake in Chile impacted intertidal invertebrates on sandy beaches along the Pacific Ocean coast [4].

In addition to intertidal settings, subtidal ecosystems, such as coral reefs, are also significantly affected by tsunami waves. The highest intensity of coral damage resulting from the 2004 tsunami in Thailand occurred in water depths of 10–20 m [5], [6]. Subsequently, initiation of coral recruitment and recover was recorded a year after the 2004 tsunami [7].

Among shallow marine settings, subtidal seafloor ecosystems composed of fine-grained sediments (sand and mud) are the most heavily disturbed by tsunami waves, as they are radically altered by rapid erosion and deposition of the seafloor. For example, Chavanich et al. reported that a 2-m-thick sand layer was eroded from the seafloor by the 2004 Indian Ocean tsunami at a water depth of 30 m on a coral reef in Thailand [5]. Goto et al. investigated changes in the topography of a sandy beach in Sri Lanka before and after the 2004 Indian Ocean tsunami, and observed sediment deposition up to 4 m thick along the shoreface (surf zone) slope [8]. Furthermore, tsunami are also known to affect the grain-size composition of seafloor sediments [9]–[11].

Severe disturbance of the seafloor by tsunami suggests that animals living on and below seafloor are also seriously affected, as their habitat is buried or eroded. Nevertheless, a lack of pre-event data often hamper investigations focusing on the impact of a tsunami on subtidal benthic ecosystems, as comparisons between pre- and post-tsunami conditions are rarely possible. Large benthic animals (megabenthos) are particularly important in seafloor environments as they mix sediments, thereby influencing the biogeochemistry of seafloor sediments [12], [13]. However, such animals are rarely studied due to the difficulty of quantitative sampling in subtidal areas. As a result, the response of subtidal megabenthos to tsunami disturbance has not been clearly detailed, although some studies have reported the post-event condition of the benthos in shallow, soft-bottom environments [11].

A megathrust earthquake with M9.0 (herein referred to as the 2011 M9.0 Tohoku-Oki earthquake) occurred on 11 March 2011, rupturing the plate boundary off the Pacific coast of northeastern Japan [14]–[17]. This earthquake generated a huge tsunami that ultimately affected a 2000 km stretch of the Pacific coast of Japan [18]–[24].

Along the Sanriku Coast, a ria coast on the northern section of Honshu Island, the narrow, funnel-shaped bays amplified the tsunami waves, generating extensive run-up and inundating land to significant heights. For example, the maximum run-up height at Miyako was 39.7 m [22]. The huge tsunami in this area suggests that the subtidal seafloor ecosystem of the ria coasts was also seriously affected by the tsunami: several meters of seafloor sediments were eroded by the tsunami at water depths of 10–20 m in ria coasts, northeastern Japan [19].

To elucidate the ecological impacts of a tsunami on the nearshore zone, data on seafloor topography, seafloor sediments, and benthic fauna, detailing pre- and post-event conditions, must be obtained. However, it is extremely difficult to investigate conditions prior to an event, as megathrust earthquakes and associated tsunami are only predictable with low precision.

Many researchers have been investigating the impact of the 2011 tsunami on marine soft-bottom ecosystems, focusing on tidal flats [25]–[31] and deep seafloor environments [32]. However, to our knowledge, no study has been conducted on benthic animals inhabiting subtidal soft-bottom environments, despite the likelihood of them being the most seriously affected by the 2011 tsunami.

**Figure 1:**
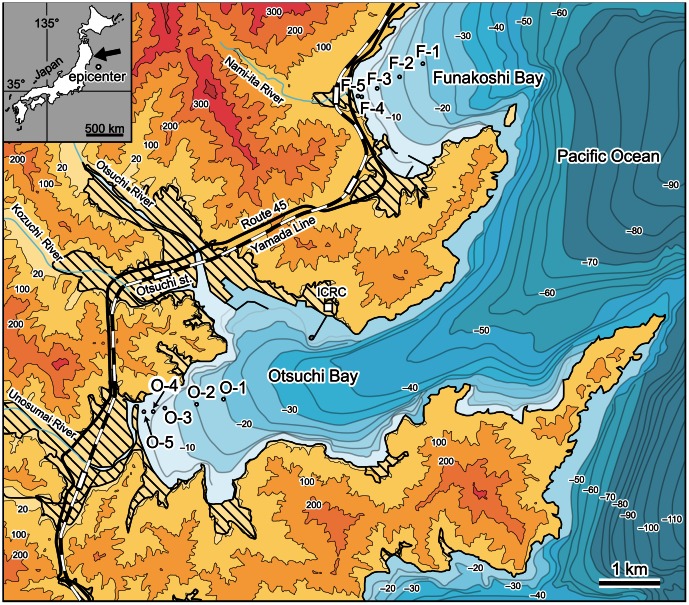
Map of the study area and sampling stations. The striped area represents tsunami inundation. Open circles represent sampling stations (O-1 to O-5 in Otsuchi Bay; F-1 to F-5 in Funakoshi Bay). ICRC: International Coastal Research Center, Atmosphere and Ocean Research Institute, the University of Tokyo. The map inset shows the epicenter of the 2011 M9.0 Tohoku-Oki earthquake and the study site (black arrow).

In this study, field surveys using scuba equipment were carried out on the seafloor of Otsuchi and Funakoshi bays, northeastern Japan (Figure 1) in 2010–2011, providing a rare opportunity to evaluate the impact of the tsunami on shallow marine seafloor topography, sediments, and megabenthos assemblages. In addition, we conducted field observations in 2012 to understand the recovery of the benthic ecosystem from the disturbance. The dataset of this study includes data obtained 6 months before the tsunami (September 2010), 6 months after the tsunami (September 2011), and 18 months after the tsunami (September 2012). Our results are the first that enable the investigation of changes and recovery in the subtidal benthic ecosystem in soft-bottom environments following a tsunami.

## Materials and Methods

### Ethics Statement

Field survey in this study is approved by local fisheries cooperative and Iwate prefecture.

### Study Sites

The Otsuchi Bay site is located in the inner part of the ria coast, and has adjacent coastal plains supporting three rivers. The Funakoshi Bay site faces the open ocean, and is therefore affected by direct ocean wave activity. In contrast to Otsuchi Bay, it has no large rivers or wide coastal plain. We established sampling stations at water depths of 20, 15, 10, 5, and 2.5 m in both bays in September 2010 (Figure 1). Previous studies conducted before the 2011 Tohoku earthquake revealed that the seafloor at depths of less than 20 m is predominantly covered with muddy and sandy deposits at Otsuchi Bay and Funakoshi Bay, respectively [33]–[35]. Sediment on the river bed in the Otsuchi area is composed of coarse-grained sands and gravels [34].

### The 2011 Tsunami Wave in the Study Area

Thirty minutes after the 2011 M9.0 Tohoku-Oki earthquake, offshore tsunami waves over 6 m high were recorded by GPS buoys drifting on the sea surface in the study area [21]. Water depth at this location is 204 m. Some papers reported tsunami inundation and run-up along the Pacific coast [22], [24]. The narrow bays along the ria coast focused the tsunami waves, generating high inundation and run-up. The behavior of the tsunami on land showed clear regional dependence, even between the adjacent bays examined in this study. At Otsuchi Bay, the maximum height of tsunami inundation and run-up were 14.7 and 19.4 m above mean sea level, respectively. The maximum run-up distance from the coastline was 3.7 km. In contrast, at Funakoshi Bay, the maximum height of tsunami inundation and run-up were 22.1 and 29.3 m above mean sea level, respectively, and the maximum run-up distance was 1.1 km from the coastline. The amount of coseismic subsidence of the ground in this area was 0.58–0.62 m [36]. Figure 1 shows extensive damage to coastal plains induced by the tsunami; the beach located at the river mouth in Otsuchi Bay was completely inundated and eroded by the tsunami, similar to other beaches along the Pacific coasts [37].

### Sampling of Megabenthos and Sediments

We recorded the presence or absence of each species of megabenthos (defined here as >2 cm in size); however, we did not collect quantitative data regarding benthos distribution metrics such as spatio-temporal change in population density and body size of the animals.

**Figure 2:**
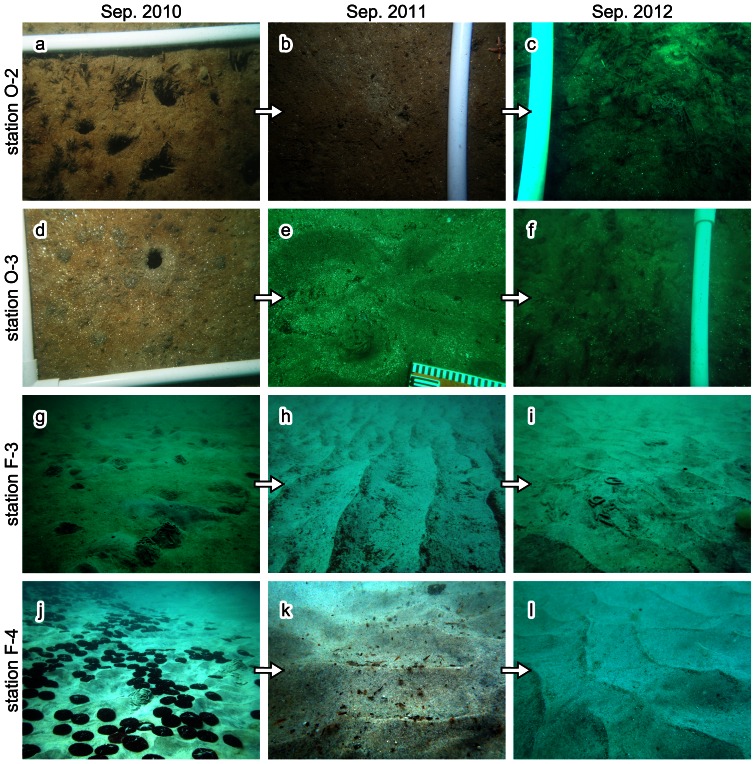
Seafloor photographs from representative sampling stations. Left, middle, and right were taken in September of 2010, 2011, and 2012, respectively. The plastic pipe provides a scale and is 2 cm in diameter. **a–c**: Seafloor at station O-2. Note the demise of large burrow openings since 2011 (after the tsunami). **d–f**: Seafloor at station O-3. The muddy seafloor changed to a sandy bed after the tsunami, and then reverted to a muddy bed. **g–i**: Seafloor at station F-3. Note the decrease in the number of fecal mounds produced by the lugworm *Arenicola brasiliensis* after the tsunami. The fecal mounds are approximately 10 cm in diameter. Ripple spacing in h and i is approximately 20 and 10 cm, respectively. **j–l**: Seafloor at station F-4. Note the complete demise of the sand dollar *Scaphechinus mirabilis* (black objects on seafloor) after the tsunami. The disc of the echinoderm is approximately 5 cm in diameter. Ripple spacing in k and l is 5–10 cm.

Sampling of megabenthos on and/or in seafloor sediments was conducted during scuba diving expeditions in September of 2010, 2011, and 2012. We excavated sediments up to 20 cm deep by hand, and collected emergent benthic animals. The sampling was conducted for more than 20 minutes at each station by two researchers. We recorded recovered species as a ‘present species’ for each station and date. We describe only abundantly recovered species (more than 3 individuals from a single station during the study period), as less abundant animals do not provide sufficient data for accurately assessing changes in spatio-temporal distribution. In addition, mobile animals such as crabs, hermit crabs, and starfish were disregarded as they are able to migrate easily between different environments (e.g., between intertidal and deeper zones of soft and rocky substrate). Sampling of deep-burrowing infauna is problematic due to the decimeter-deep sediments under which they dwell. Biogenic sedimentary structures such as burrow and fecal mounds enable us to recognize their presence. On the basis of underwater field surveys, we confirmed that spiral fecal mounds in the study area are produced by the lugworm *Arenicola brasiliensis* (Polychaeta: Arenicolidae). As this lugworm is rarely recovered during benthos sampling, we recorded the presence or absence of fecal mounds (Figure 2g) to identify its distribution.

Seafloor sediments were also collected from each sampling station at each sampling date. In the laboratory, median grain size (*D_50_*) and mud content (%) were determined for these sediment samples using conventional sieving methods. Water depth was recorded during each sampling period using a dive computer (Suunto Gekko, accuracy of water depth observation: 0.1 m) at each sampling station. Measured water depth value was calibrated to depth from reference level (mean sea level) using tide table. Seafloor photographs were taken to record changes in environmental conditions at the sampling stations.

## Results

### Changes in Environmental Conditions of the Seafloor

During the study period, our results show episodic changes in topography and grain-size composition in the study area (Table 1). Representative seafloor photographs are shown in Figure 2.

**Table 1:** Environmental parameters of seafloor for each sampling station and date.

			Water depth (m)			Median grain size *D*_50_ (mm)			Mud contents (%)		
Station	Latitude (N)	Longitude (E)	2010-Sep.	2011-Sep.	2012-Sep.	2010-Sep.	2011-Sep.	2012-Sep.	2010-Sep.	2011-Sep.	2012-Sep.
O-1	39°20.320’	141°54.864’	20.6	22.2	21.5	0.0424	0.1340	0.0774	74.7	17.6	48.2
O-2	39°20.276’	141°54.564’	15.9	15.8	16.1	0.0535	0.0736	0.1104	71.0	51.3	23.5
O-3	39°20.244’	141°54.209’	10.2	8.2	9.9	0.1149	0.5643	0.0678	29.2	1.9	53.9
O-4	39°20.212’	141°54.079’	4.7	5.5	5.9	0.1748	0.4558	0.4790	1.6	0.3	1.5
O-5	39°20.221’	141°53.977’	1.8	2.7	2.6	0.2501	0.3689	0.3309	0.2	0.3	0.1
F-1	39°23.247’	141°57.121’	20.4	21.0	20.7	0.1191	0.1375	0.1430	5.9	4.6	9.6
F-2	39°23.135’	141°56.861’	15.9	16.5	16.1	0.1274	0.1306	0.1400	6.6	11.3	6.2
F-3	39°23.036’	141°56.611’	10.5	12.7	11.4	0.1239	0.1199	0.1096	11.1	7.8	15.8
F-4	39°22.966’	141°56.438’	4.9	5.1	5.8	0.1751	0.1870	0.1513	3.4	1.5	2.2
F-5	39°22.970’	141°56.397’	3.1	3.6	4.1	0.2178	0.2099	0.1540	0.0	0.5	8.3

Between 2010 (pre-tsunami) and 2011 (post-tsunami) at Otsuchi Bay, water depth increased at stations O-1, O-4, and O-5 as seafloor sediments were eroded. Conversely, water depth decreased at O-2 and O-3 due to the deposition of a coarse-grained sand layer. An almost complete reversal of these bathymetric changes was observed in stations O-1 and O-3 between 2011 and 2012.

In Otsuchi Bay, seafloor sediment changed from muddy to coarse-grained sand between 2010 and 2011, except at station O-2. However, in the 2012 sample, grain-size composition had almost returned to pre-tsunami conditions, with the exception of station O-4. The most prominent example of this recovery was seen at station O-3 (Table 1; Figure 2d–f). Mud content in the sediment samples also decreased after the tsunami, and then increased between September 2011 and September 2012.

At Funakoshi Bay, changes in water depth and sediment grain size were much less marked than in Otsuchi Bay. Station F-3 showed significant topographical change, but changes at all other stations were below the level of maximum seismic subsidence (0.6 m) in this area.

Seafloor photographs also show changes in small-scale seafloor morphology (Figure 2). For example, invertebrate burrows with openings greater than 1 cm in diameter were abundant at stations O-2, O-3, and F-3 in 2010, and physical sedimentary structures such as ripple marks were not visible before the tsunami as a result of bioturbation (Figure 2a, d, g). Six months after the tsunami (September of 2011); however, large burrow openings had virtually disappeared (Figure 2b, e, h) and the seafloor was covered with physical sedimentary structures. In September 2012, the number of large burrows had increased slightly (Figure 2c, f, i).

### Change in Megabenthos Distribution

A total of 9 abundantly recovered species were analyzed in this study, although other 6 less abundant animals were also recognized. The results of benthos sampling showed four succession patterns in the benthos distribution: Pattern I, disappearance of a species after the tsunami; Pattern II, constant presence of a species before and after the tsunami; Pattern III, disappearance of a species 6 months after the tsunami (September 2011) followed by reappearance 18 months after the tsunami (September 2012); and Pattern IV, absence of a species before the tsunami but presence following the tsunami.

### Otsuchi Bay

**Figure 3:**
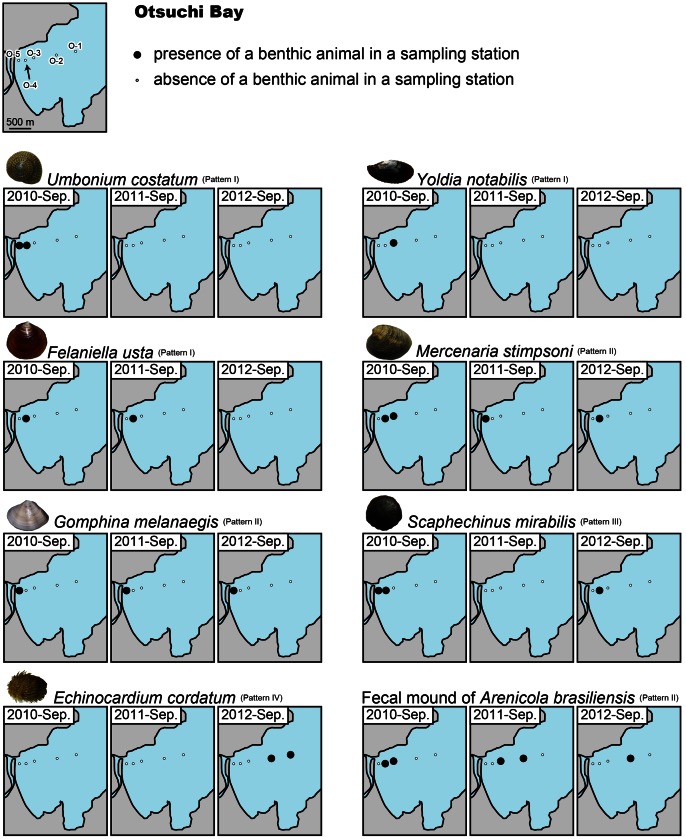
Results of megabenthos sampling in Otsuchi Bay. Large solid circles represent the presence of a benthic animal in a sampling station, while small open circles represent absence. Left, middle, and right maps represent the distribution of each species in September of 2010, 2011, and 2012, respectively. Note that the distribution succession differs among the species.

Figure 3 depicts the results of benthos sampling at Otsuchi Bay. Animals following Pattern I include the giant button top shell *Umbonium costatum* (Gastropoda: Trochidae), the protobranch bivalve *Yoldia notabilis* (Bivalvia: Nuculanidae), and the heterodont bivalve *Felaniella usta* (Bivalvia: Ungulinidae). Pattern II was observed for the venus clam *Mercenaria stimpsoni* (Bivalvia: Veneridae). Based on observations of the characteristic fecal mound, the lugworm *Arenicola brasiliensis* (Polychaeta: Arenicolidae) also follows Pattern II. The presence of the venus clam *Gomphina melanaegis* (Bivalvia: Veneridae) was consistent with Pattern II succession. In addition, the location over which this bivalve was distributed remained unchanged throughout the study period. Pattern III was observed for the sand dollar *Scaphechinus mirabilis* (Echinoidea: Scutellidae). The heart urchin *Echinocardium cordatum* (Echinoidea: Loveniidae) followed Pattern IV; it was not recovered in samples collected in 2010 and 2011, but was present in 2012, 18 months after the 2011 tsunami.

### Funakoshi Bay

**Figure 4:**
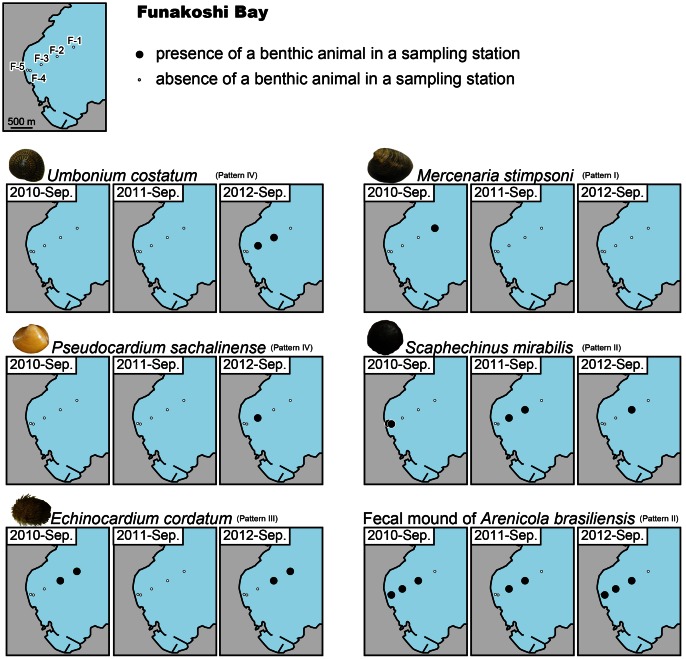
Results of megabenthos sampling in Funakoshi Bay. Large solid circles represent the presence of a benthic animal in a sampling station, while small open circles represent absence. Left, middle, and right maps represent the distribution of each species in September of 2010, 2011, and 2012, respectively. Note that the distribution succession differs among the species.

Figure 4 shows the results from samples collected in Funakoshi Bay. The number of species recovered from samples in this bay was lower than that in Otsuchi Bay. Echinoids and polychaete were dominant in this area. *M. stimpsoni* was the only species characterized by Pattern I at this location. Benthos showing Pattern II succession included *S. mirabilis* and *A. brasiliensis*; however, the details of this succession were not consistent between these two species. The distribution of *S. mirabilis* experienced a general migration toward the offshore stations after the tsunami. In contrast, the location of *A. brasiliensis* showed relatively little change in the immediate aftermath of the tsunami, and had returned to the pre-event situation by September 2012. Although the available distribution data do not reveal changes in population density, seafloor photographs clearly show decreasing density of echinoderms and fecal mounds of polychaete at the shallow stations following the tsunami (Figure 2j–l for *S. mirabilis*; Figure 2g–i for *A. brasiliensis*). The heart urchin *E. cordatum* followed Pattern III, disappearing after the tsunami and then reappearing in similar locations in the 2012 samples. The Sakhalin surf clam *Pseudocardium sachalinense* (Bivalvia: Mactridae) and the giant button top shell *U. costatum* showed Pattern IV succession.

## Discussion

### Impact of the 2011 Tsunami on the Seafloor Environment

The present results do not reveal detailed temporal changes in the seafloor environment, as we only investigated five sampling stations along a single transect for each bay. Nevertheless, this study can provide important information for understanding the impact of the tsunami on shallow marine soft-bottom settings, as it provides a rare insight into changes caused by an episodic event.

Our results from water depth measurements and analyses of seafloor sediment grain-size composition in 2010 (six months before the tsunami) closely correspond to those reported in studies conducted in this area in the 1980s [33], [34]. In contrast, these parameters showed considerable change between 2010 and 2011, especially in Otsuchi Bay. This indicates that the seafloor environment had been stable for more than 30 years, before being radically disturbed by the 2011 tsunami. Such dramatic changes in bathymetry and grain-size composition in nearshore zones following tsunami have also been reported by previous studies, although many lack data from immediately prior to the event. Chavanich et al. and Goto et al. reported radical erosion and deposition, respectively, resulting from the 2004 Indian Ocean tsunami [5], [8]. Radical changes in seafloor bathymetry by the 2011 tsunami have been also reported from the other ria coasts of north eastern Japan [19]. Tsunami are also known to alter the grain-size composition of seafloor sediments [9]–[11]. Hence, bathymetric and sediment changes can be considered a consistent feature of tsunami disturbance in nearshore environments.

Care should be taken when interpreting the changes in bathymetry and grain size at stations in depths of less than 10 m (the shoreface environment). This includes stations such as O-4, O-5, F-4, and F-5. The shoreface (surf zone) represents a physically unstable environment; its seafloor topography and sediments frequently change through migration of longshore bars in response to wave conditions (i.e., shoreface morphodynamics [38]–[41]). Therefore, the changes observed between the 2010 and 2011 measurements may not accurately represent the tsunami impact. Subsequent sediment dynamics may have overprinted or erased the changes induced by the 2011 tsunami on the seafloor at the shallow stations.

Changes in the seafloor environment in Otsuchi Bay were more substantial than those in Funakoshi Bay. In the former, the average grain size of sediment increased from 2010 to 2011. Muddy substrate was replaced by a coarse-grained sand layer, inducing seafloor aggradation to a maximum height of 2 m. Prior to the tsunami, the seafloor of the inner area of Otsuchi Bay, close to the river mouth, was covered with muddy deposits, while sediment on the river bed was composed of coarse-grained sand and gravels [33], [34]. The maximum tsunami run-up distance from the coastline was 3.7 km in Otsuchi Bay. We interpret the changes in grain-size distribution following the tsunami as a result of seawater that flooded the coastal plain backwashing to the ocean through the rivers. This would result in a considerable amount of river bed sediments and beach deposits being transported to the subtidal area. This interpretation is supported by the complete inundation and erosion of the sand spit near the mouth of the Unosumai River after the tsunami, and the presence of coarser sediment at the shallow stations (O-3, -4, -5 in 2011). The most significant difference in the terrestrial environments of Otsuchi and Funakoshi bays is the existence of coastal plains and rivers in Otsuchi Bay that supplied coarse-grained sediments. The seafloor of Funakoshi Bay may have remained unchanged due to a lack of coarse-grained terrestrial sediments in the area. Therefore, the impact of tsunami on the shallow marine seafloor appears to depend on the local terrestrial environment.

Considering the maximum coseismic subsidence of 0.6 m in this region [36], the water depths at stations F-1 and F-2 remained superficially unchanged between 2010 and 2011 (Table 1). Furthermore, the grain-size distributions at these stations remained largely unchanged. However, animals inhabiting the sediment, such as *E. cordatum*, disappeared before the 2011 samples were collected, suggesting that the seafloor had been disturbed by the tsunami to some extent.

Goto et al. suggested that the impact of tsunami on permanent geomorphological landforms was considered to be limited in shallow marine settings [8]. Despite observing considerable sedimentation on a beach in Sri Lanka from the run-up of the 2004 Indian Ocean tsunami, they reported that landforms generated by the tsunami had undergone almost complete reversion to their pre-tsunami condition. The results of the present study are consistent with this phenomenon. For example, the water depth at station O-3 showed a decrease in 2011 due to deposition of a coarse-grained sand layer. However, by September 2012, the water depth and grain-size composition were approximately the same as they were in 2010. This indicates that the seafloor environment in the study area is reverting to its pre-tsunami condition. As outlined previously, our data are not of sufficient spatio-temporal resolution to identify the mechanisms driving this reversion.

### Impact of the 2011 Tsunami on the Megabenthos and their Subsequent Recovery

As described above, the results of this study only provide qualitative data on the megabenthos distribution at a few sampling stations along a single transect in each bay. Furthermore, the data do not include changes in the distribution of small benthic animals less than 2 cm in size, and as such cannot detail larval settlement during the study period. Nevertheless, the data provide clues to the impacts of tsunami on soft-bottom ecosystems. Although some studies have described the seafloor benthos assemblages after tsunami [11], the present study is the first to directly compare the pre- and post-tsunami subtidal benthic assemblages. Megabenthos reaching more than several centimeters in size plays an important role in the seafloor ecosystem [12], [13]; however, they are rarely recovered using quantitative sampling methods such as grab sampling from ships. Our results obtained from underwater observation therefore provide useful information on large benthic animals.

Other than the lugworm *A. brasiliensis*, all megabenthos described in this study have been observed in Otsuchi and Funakoshi bays since the 1970s (Table 2). This indicates that the megabenthos assemblage has been stable for more than 30 years. However, some benthic animals disappeared after the 2011 tsunami, synchronous with changes in the seafloor environment. This indicates that disturbance by the 2011 tsunami severely impacted the megafaunal assemblage in the soft bottoms of the ria coasts.

**Table 2:** Summary of published information on magabenthos in the study sites.

Species	Locality	References
*Umbonium costatum*	Otsuchi Bay	[49], [50], [51]
*Yoldia notabilis*	Otsuchi Bay	[42], [43], [49], [50], [52], [53]
*Felaniella usta*	Otsuchi Bay	[50], [54]
*Mercenaria stimpsoni*	Otsuchi Bay	[50], [55]
*Gomphina melanaegis*	Otsuchi Bay	[50], [55]
*Pseudocardium sachalinense*	Otsuchi Bay	[50]
*Scaphechinus mirabilis*	Otsuchi Bay	[49], [50], [52], [54]
*Echinocardium cordatum*	Otsuchi Bay	[50], [52], [54]
	Funakoshi Bay	[33]

The differing patterns of succession and distribution for each species following the tsunami indicate that the impact of tsunami is species-dependent. In addition, different patterns of succession were observed for a single species in the two bays; e.g., *E. cordatum* and *U. costatum*. Therefore, the impact of tsunami on the megabenthos assemblage is dependent on environmental conditions as well as species.

The demise of animals with a succession following Pattern I is interpreted as an inability to adapt to rapid sedimentation and/or changes in seafloor environment (grain-size composition) following the tsunami. For example, *Y. notabilis* normally inhabits hydrodynamically calm muddy sediments on the seafloor [42], [43], which experience little erosion or deposition. This means that the bivalve is rarely exposed to rapid topographical changes. During the 2011 tsunami, the muddy seafloor was buried beneath 2-m-thick coarse-grained deposits, to which it was unable to adapt. It should be noted that our observation covers limited areas of the bays, and animals may have survived in ‘refuge areas’ in other parts of the ria coast.

In Funakoshi Bay, the succession of *S. mirabilis* followed Pattern II. However, *S. mirabilis* disappeared from its original habitat, and its distribution moved offshore. This change in distribution occurred because *S. mirabilis* inhabits the exposed seafloor, and is not buried under seafloor sediments (Figure 2j). It is therefore easily transported by water currents, such as those induced by the 2011 tsunami.

In Otsuchi Bay, *G. melanaegis* also showed Pattern II succession, but the spatial distribution of the species did not change throughout the study period (Figure 3). This bivalve is known to live on longshore sand bars in the surf zone of sandy beaches facing the open ocean [44]. These bars constantly migrate in response to changing wave conditions, frequently generating meter-scale erosion and deposition on the seafloor [38]–[41]. *G. melanaegis* and other infauna inhabiting high-energy sandy beaches are known to have unique behavior for adapting to topographical changes [44]–[47]. These abilities are likely to have allowed *G. melanaegis* to survive topographical changes caused by the tsunami.

Although the bathymetry in Funakoshi Bay showed a slight change following the tsunami, fecal mounds of *A. brasiliensis* were evident in the bay throughout the study period. This indicates that the polychaete was also able to adapt to topographic changes induced by the tsunami. However, the extent of the spatial distribution of mounds diminished following the tsunami in Otsuchi Bay, where both bathymetry and sediment grain size showed marked changes. This evidence suggests that *A. brasiliensis* may be unable to adapt to changes in grain-size composition of the seafloor and/or large-scale changes in topography.

A similar succession to that described by Pattern III was reported after investigating offshore benthic fauna on Thailand’s Andaman Coast following the 2004 Indian Ocean tsunami [11]. Although their data lack pre-tsunami information, they described an abundance of the heart urchin *Brissopsis luzonica* (Echinoidea: Brissidae) in November 2007. This was interpreted as a population originating from post-tsunami recolonization of the disturbed seafloor [11]. The results of the present study also show a complete demise of the heart urchin *E. cordatum* in Funakoshi Bay in 2011, followed by recolonization of the bay at 18 months after the tsunami. Reestablishment of these echinoderm populations therefore occurred within a few years of disturbance following both the 2004 Indian Ocean tsunami and the 2011 M9.0 Tohoku-Oki earthquake tsunami.

The presence of *E. cordatum* in Otsuchi Bay followed Pattern IV, as it was only recovered in samples from September 2012. This suggests new colonization of the disturbed seafloor by the species. At shallower stations in Funakoshi Bay, *P. sachalinense* and *U. costatum* were also present only in the 2012 samples. Therefore, the tsunami disturbance appears to have changed the composition of the megafaunal community in the ria coasts. However, the mechanism by which this shift in community composition occurred is unclear. As detailed earlier, the method we used for sampling provides no information on the distribution of the meiobenthos or macrobenthos, and consequently cannot reveal larval settlement patterns after the 2011 tsunami. Species showing Pattern IV succession must have originated from new larval settlements. Future studies will investigate the detailed mechanism of megabenthos recolonization of the tsunami-disturbed seafloor using cohort analysis.

The 2011 tsunami impacted the macrofaunal assemblage in the soft-bottomed coasts, but the changes in the megabenthos distribution were not uniform: they varied for each species, and were dependent on the environmental conditions in the bays we studied. Such differences may originate from the ecological niche of each species, and are likely to affect the recovery process of the seafloor ecosystem following tsunami disturbance.

Huge tsunami induced by megathrust earthquakes have recurred at intervals of 500–800 years in northeastern Japan [48]. Therefore, these waves must periodically affect the coastal ecosystems. Accordingly, the seafloor ecosystem in the study area has repeatedly experienced disturbance by tsunami in the past. Continued monitoring in the future is vital to understanding the nature of the succession of the benthic assemblages in the area, and for assessing the long-term effect of tsunami waves on the megabenthos assemblage.
